# Prevalence of Reproductive Health Issues among US Female Law Enforcement Officers

**DOI:** 10.3390/healthcare11192647

**Published:** 2023-09-28

**Authors:** Ainslie Kehler, Sara Jahnke, Filip Kukić, Aspen E. Streetman, Katie M. Heinrich

**Affiliations:** 1 Department of Kinesiology, Kansas State University, Manhattan, KS 66506, USA; aestreetman@ksu.edu (A.E.S.); kmhphd@ksu.edu (K.M.H.); 2 National Development & Research Institutes, Leawood, KS 66224, USA; sara@hopehri.com; 3 Faculty of Physical Education and Sports, University of Banja Luka, 78000 Banja Luka, Bosnia and Herzegovina; filip.kukic@gmail.com

**Keywords:** police officers, pregnancy, miscarriage, preterm birth, occupational health

## Abstract

Reproductive health is a considerable concern among US female law enforcement officers (LEOs). Miscarriage and preterm birth rates are significantly higher in women firefighters than published US averages. Since law enforcement and firefighting share occupational conditions and practices, adverse birth outcomes were hypothesized to be greater in female law enforcement officers (LEOs) than the US averages. Occupational hazards may place pregnant LEOs at a higher risk for complicated pregnancies and adverse birth outcomes. This study quantified pregnancy outcomes in female LEOs using a cross-sectional survey and compared them to US averages and large prospective studies. The participants (N = 162, 72.2% aged 31–49, 85.2% Caucasian) averaged 2.5 ± 1.4 pregnancies. Stress (59.1%) and shiftwork (59.8%) were the most common reported exposures. Miscarriage and preterm birth rates were 19.1% and 16.4%, respectively. Miscarriages were significantly greater among participants compared to prospective studies [χ^2^ (1, N = 911,971) = 20.51, *p* < 0.001]. Female LEOs of childbearing age should receive education about potential reproductive health hazards and take precautions against them. Moreover, policymakers, human resources, and healthcare providers should understand how law enforcement work might affect maternal health.

## 1. Introduction

A career in law enforcement is inherently stressful and high-risk compared to other careers [1]. Law enforcement officers (LEOs) are regularly exposed to confrontation, potential harm, and violence [2]. LEOs report physical (e.g., cardiovascular and musculoskeletal disorders) and psychological health symptoms (e.g., posttraumatic stress disorder, depression) due to occupational stress [3,4,5,6].

Law enforcement is a male-dominant occupation since female officers comprise only 12.6% of sworn officers in the US [7]. Female LEOs experience more stress than their male counterparts. Stress sources include discrimination, harassment, and adapting to a male-dominated profession, which may increase job stress [8]. However, female LEOs are an asset to law enforcement. For example, they are more effective in responding to incidents of violence against other females than male LEOs [9], and they are less likely to use extreme controlling behaviors during arrests (e.g., threats, physical restraint) than male officers [10]. Efforts to increase the recruitment and retention of female LEOs have been largely unsuccessful, perhaps because female LEOs’ reproductive health needs are not addressed by policing organizations [11].

Female LEOs encounter a myriad of occupational risk factors that may cause adverse pregnancy and birth outcomes. Shift work, high stress, allostatic load, and environmental exposures are risk factors for adverse fetal outcomes [12,13,14,15,16,17,18,19,20,21]. While these conditions are expected in law enforcement, adverse birth outcomes have never been quantified in this population, and few studies have explicitly examined female LEOs’ reproductive health. While recent research among a similar tactical occupation (firefighters) described high reported miscarriage rates (27%) [22], the dearth of research regarding female LEOs’ reproductive health is unsettling.

The US Bureau of Labor Statistics Standard Occupational Classifications [7] lists law enforcement as a shift work occupation falling under the protective service branch. About 50% of law enforcement personnel work shifts [23]. Shift work disrupts circadian rhythms, negatively impacting physiological function [17] and changing hormonal concentrations that affect conception and normal fetal development [15]. Shift work’s ill effects seem to compound when officers work consecutive night shifts [24] or encounter short intervals between night shifts (i.e., quick return) [21]. Negative shift work repercussions include increased anxiety, depression, neuroticism, gastrointestinal disorders, weight gain, type II diabetes, coronary heart disease, and pregnancy hypertensive disorders [25,26]. Shift work schedules may also increase miscarriage, low birth weight risk in infants, and preterm delivery risks [12,19,21].

High job stress has been associated with an increased risk of miscarriage and low birth weight among females [13,27]. The allostatic load of law enforcement work wears the body down from prolonged exposure to stress, increasing stress-response system activation [28,29,30]. The cumulative pathway model shows that allostatic load has been connected to declines in health and other negative health outcomes, including adverse birth outcomes [28,29,30]. Thus, it is logical to think female LEOs are at a heightened risk for the adverse effects of allostatic load due to prolonged and consistent exposure to occupational stress.

Workplace exposures to chemicals and disease-causing agents can affect a female’s reproductive health, including childbearing ability and fetal health and development [14]. Chemical exposures can lead to reproductive health problems such as infertility/reduced fertility, menstrual/ovulatory cycle disorders, sex hormone imbalances, miscarriage, stillbirth, congenital disabilities, child developmental disorders, premature birth, or lower-birth-weight babies [14]. During the first trimester, exposure to harmful substances increases risks for a congenital disability or miscarriage, while exposure during the second and third trimesters could result in the delayed growth of the fetus, premature birth, or impacts on brain development [31]. LEOs risk coming into contact with people carrying disease-causing agents that are known reproductive hazards, including cytomegalovirus, hepatitis B, human immune-deficiency virus, human parvovirus B19, and varicella-zoster virus [31]. LEOs face lead exposure via handling lead-based ammunition [32], leading to adverse birth outcomes, including miscarriage, preterm delivery, premature membrane rupture, and neurobehavioral effects in infants [16]. Female LEOs may also encounter other environmental exposures, such as whole-body vibrations and loud noises (e.g., weapon discharges, sirens, traffic noise, and barking police dogs) [33,34] that may negatively affect pregnancy outcomes and lead to preeclampsia, gestational hypertension, and gestational diabetes [33].

Despite the well-documented occupational risks, data for birth outcomes among female LEOs are lacking. Indeed, to our knowledge, no study has quantified birth outcomes among female LEOs in the United States. Understanding birth outcomes can help identify potential occupational health and safety risks associated with their jobs. This information can be used to develop strategies and policies to protect the health and well-being of these officers during pregnancy and childbirth. Therefore, this study aimed to provide preliminary data on birth outcomes and highlight key risk factors for female LEOs’ reproductive health. Adverse birth outcomes such as miscarriage and preterm birth were hypothesized to be significantly higher among female LEOs than in two sizeable general population birth outcome studies [35,36].

## 2. Materials and Methods

### 2.1. Participants

A cross-sectional survey was disseminated using snowball sampling techniques [37]. Inclusion criteria were females currently serving in any branch of US law enforcement aged 18–65 with at least one pregnancy during their service. Police departments did not readily provide personal contact information for officers. Primary sample recruitment began with the International Association of Women Police and the National Strength and Conditioning Association Tactical facilitator’s e-mail list and social media sites such as LinkedIn, law enforcement organizations for females, and females in law enforcement groups on Facebook. Participants were asked to refer other female colleagues interested in participating. The first page of the survey served as the informed consent document. It informed participants about the research study’s scope and purpose, anonymous and voluntary nature, and provided contact information for the principal investigator. The study was approved as exempt by the university’s Institutional Review Board (IRB #8737). Thus, formal consent was not required, and participants who proceeded to the survey’s first question opted into the study voluntarily.

### 2.2. Measures

The 78-question self-report survey was formatted similarly to female firefighter research [22]. The survey was created using Qualtrics (Provo, Utah), a secure online platform. It began with eligibility, informed participants about the study’s aims and scope, and then asked them to enter their current height and weight, pregnancy questions, and demographics. A debriefing statement was presented at the end of the survey. Participants were redirected to a separate survey to enter their e-mail into a drawing for one of ten $25 gift cards.

General demographic questions included marital status, ethnicity, income, education attainment, and occupational information such as rank and years of service. Data about pregnancy outcome, duration, maternal age at conception, and if the pregnancy occurred while employed in law enforcement were collected. Participants answered detailed questions about each pregnancy, indicating specific job exposures during pregnancies (e.g., strenuous physical demands, shift work, stress, and lead handling) and the pregnancy outcome. Each reported live birth was followed with yes/no questions to determine if the baby was born light for gestational age (≤5 lbs. 8 oz), was preterm (born ≤ 37 weeks gestational age), was diagnosed as jaundice, or had failure to thrive (inadequate growth based on anthropometrical parameters) [38]. The average time for survey completion was 18.7 min.

Birth outcomes were categorized as live birth, miscarriage, stillbirth, intended termination, still pregnant, or other. Ectopic pregnancies (n = 2) and anembryonic pregnancies (n = 1) were classified as miscarriages since these pregnancies resulted in a loss before 20 weeks and fit the miscarriage definition. We used the term miscarriage since it was the recommended terminology for pregnancy loss before viability before the mid-second trimester [39] and because families preferred miscarriage to spontaneous abortion due to negative emotional connotations [40]. Current and/or intentionally terminated pregnancies were excluded from the analysis for that pregnancy due to unknown reproductive outcomes. Since few participants had four or more pregnancies, they were combined into a single category for analysis.

### 2.3. Statistical Analysis

Frequencies of all pregnancy outcomes were calculated. Miscarriage rates were calculated for those who reported actively working as an LEO during pregnancy. Next, trends in maternal age were examined by comparing two groups: <35 years and ≥35 years (i.e., “advanced maternal age” often used for determining prenatal screening protocols) [41].

Miscarriage definitions and classifications were aligned and age-standardized to a prospective linkage study that included maternal age and fetal outcomes for >1,000,000 pregnancies [36]. Ectopic pregnancies were counted in miscarriage data, but stillbirths were not due to their definition in gestational age (pregnancy loss occurring at 20+ weeks [36]. Preterm births were standardized and compared with Blencowe et al. [35], a systematic review study with a large US sample.

## 3. Results

### 3.1. Sample Characteristics

**Figure 1:**
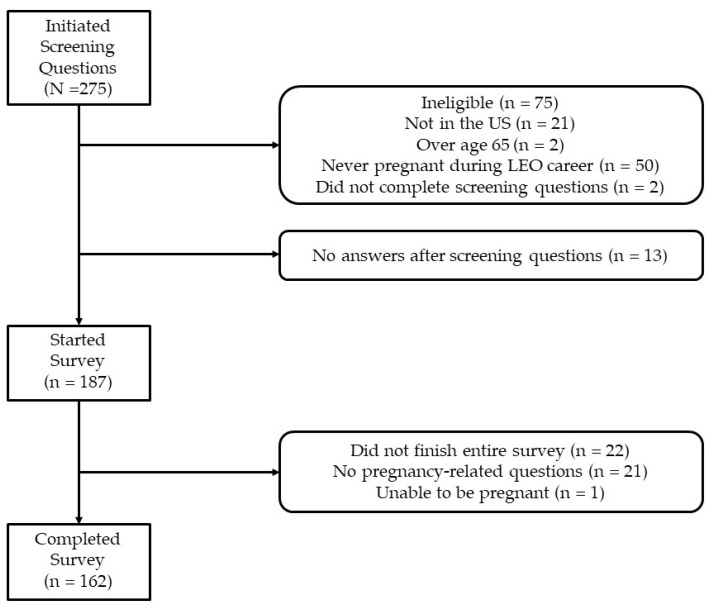
Flowchart of participants in the survey.

A total of 275 individuals clicked on the survey link (Figure 1). Of those, 21 did not currently serve in US law enforcement; two were older than 65, and 50 had never had a pregnancy during their law enforcement career. Two did not complete the screening questions, and 13 had no answers after the screening questions, leaving 187 (68.0%) eligible to complete the survey. A total of 22 individuals did not finish the survey, 2 did not complete any pregnancy questions, and 1 participant reported being unable to become pregnant, thus leaving 162 (58.9%) with complete data for analysis.

The majority (n = 138, 85.2%) were Caucasian, 13 (8.0%) were Hispanic, Latina, or of Spanish origin, 8 (4.9%) were Black or African American, and 3 (1.9%) were American Indian or Alaska Native. The largest respondent group was aged 31 to 40 (see Table 1), while 10 (6.1%) did not provide their age. The average body mass index (BMI) was 28.9 ± 5.7 kg/m^2^, with 56 (34.4%) classified as obese (BMI ≥ 30 kg/m^2^). The participants reported 402 pregnancies, averaging 2.5 ± 1.4 pregnancies per participant. Most participants (n = 116, 71.2%) were married or with a partner, 89 (54.6%) reported annual household incomes of USD 100,000+, and 96 (58.9%) reported completing college or advanced degrees. The participants were from 32 of 50 states. The states with the most prominent response rates were New York (n = 22, 13.5%), Nebraska (n = 15, 9.2%), Wisconsin (n = 13, 8.0%), California (n = 12, 7.4%), and Kansas (n = 12, 7.4%).

### 3.2. Occupational Information

More participants had served in law enforcement for 11–15 years (n = 45, 27.8%) and FOR 20+ years (n = 44, 27.2%; see Table 2). The largest respondent group reported being an officer (police, patrol, constable, or deputy sheriff), followed by detectives/investigators and sergeants. Half described their department as urban, compared to suburban or rural. Fifty participants (30.9%) reported that their spouses also worked in law enforcement.

### 3.3. Pregnancy Outcomes

Of the 402 pregnancies analyzed for the 162 participants, 6 were currently pregnant, and 2 reported having stillbirths, leaving 394 pregnancies for analysis. Overall, pregnancy outcomes included 293 live births (74.4%), 69 miscarriages (17.5%), and 32 terminations (8.1%). The miscarriage rate was higher among those ≥35 years (24.7%) than those <35 years (15.9%). The age-adjusted pregnancy outcomes are presented in Table 3. The age-adjusted miscarriage rates were higher than the Andersen et al. [36] sample, which reported a miscarriage rate of 12.0% in females under 35 and 28.3% in females ≥35 years. The proportion of overall miscarriage rates compared to live births was higher than the Andersen et al. [36] sample [χ^2^ (1, N = 911,971) = 20.51, *p* < 0.001]. LEOs under the age of 35 and ≥35 years had significantly higher miscarriage rates than the Andersen sample at χ^2^ (1, N = 865,472) = 10.72, *p* < 0.001.

### 3.4. Maternal and Child Health

Of the 293 live births, 6.5% (n = 19) were light for gestational age (see Table 4). Almost one-third (29.7%, n = 87) had jaundice, and 2.7% (n = 8) failed to thrive. Sixteen percent (n = 47) were preterm (born three weeks before their due date). Preterm births in our LEO sample were >4% higher than in the Blencowe et al. [35] sample (12.0%). Self-reported preterm births were significantly higher in our sample than in Blencowe [35] [χ^2^ (1, N = 4,300,935) = 5.15, *p* = 0.02]. In 15.5% (n = 45) of pregnancies, participants were diagnosed with some form of high blood pressure and gestational diabetes in 9.6% (n = 28) of pregnancies. High blood pressure was more prevalent than diabetes when stratified by pregnancy (see Table 4).

### 3.5. Pregnancy Practices

**Figure 2:**
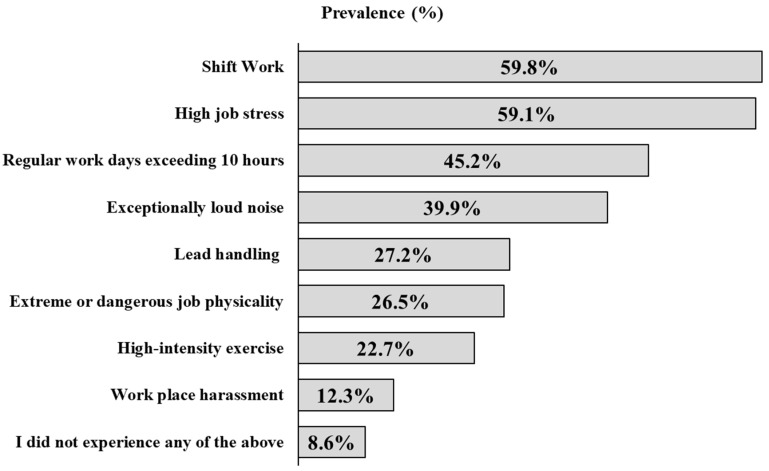
Average self-reported job exposures. Note: Exposures were calculated from the first four pregnancies only.

The participants actively responded to emergency calls 59% (n = 230) of the time after learning of their pregnancy. The participants’ most widely reported job exposure was shift work, followed by high job stress, shifts longer than 10 h, and exceptionally loud noise (see Figure 2). Less than 30% reported exposure to lead handling, extreme or dangerous physical jobs (e.g., violence, wrestling, assault, apprehension), and high-intensity exercise (e.g., sprinting, jumping, lifting, carrying). Less than 15% reported workplace harassment.

## 4. Discussion

### 4.1. Overall Discussion

This study provides novel information for birth outcomes and key risk factors among female LEOs in the US. As hypothesized, miscarriage and preterm birth rates were significantly higher than those of two large general-population birth outcome studies [35,36]. Additionally, miscarriage and preterm birth rates were higher than the American College of Obstetricians and Gynecologists (ACOG) statement that miscarriage tends to occur in 10% of pregnancies [42]. Since age is the strongest miscarriage predictor [36,43,44,45], our age-adjusted miscarriage rates provide evidence of a higher rate of miscarriage among US female LEOs compared to more than one million Danish females [36].

Few biological, socioeconomic, or behavioral factors other than increased maternal age have been consistently associated with increased miscarriage risk [36,43,44,45]. One study of Danish women identified behavioral risk factors such as alcohol consumption, working at night, and lifting over 20 kg daily [46]. In our sample, the age-adjusted miscarriage rate for female LEOs under 35 years was 17.9% and increased to 24.7% in female LEOs aged 35 and older. According to the most recent bulletin published by the ACOG, the miscarriage risk is between 9 and 17% for ages 20 to 30 years and 20% for ages 35 to 40 [42]. Thus, our findings still appear higher for both age groups.

Our study’s crude miscarriage rate was almost 6% higher than that of Andersen et al. [36], a large prospective study reporting over one million pregnancies and outcomes (19.6% vs. 13.2%). Of note, miscarriage rates differ in the literature due to classification and how pregnancy loss is measured. For example, the ACOG [42] estimates that 10% of all early pregnancies end in miscarriage. One systematic review reported that miscarriage occurs in between 11% and 22% of all pregnancies [47]. Thus, while not definitive, our results suggest miscarriage in LEOs is higher than in the general US population.

The preterm birth rate was significantly higher, occurring in 16.4% of our sample. The national preterm birth rate is between 9.8% and 12% [35,48]. Occupational exposures such as working nights [49] and prenatal lead exposure [50] increase preterm birth risk. Our sample reported shift work, including night-time shifts, in almost 20% of all pregnancies and lead exposure in 9.3% of pregnancies. It is of note that gestational diabetes and hypertension are also preterm birth risk factors [51]. Our sample’s rate of gestational hypertension was just over 11%, which is higher than the 6–8% US average [52]. However, only 7% of our sample had gestational diabetes, less than the 9% US average [53].

Over 71% of participants reported actively responding to emergency calls when they learned of their pregnancy. This was similar to Jahnke et al. [22] who found that most firefighters actively responded to calls in the same time period. The highest exposures were shift work, high job stress, and shift lengths lasting ten or more hours during pregnancy. Irregular work hours are linked with an even higher risk of miscarriage and reduced fertility, while regular work shifts, including night shifts, do not appear to have the same effect [15]. More importantly, chronic exposure to stress has been associated with increased allostatic load [28], which has also been connected to adverse pregnancy and birth outcomes [29,30]. Therefore, assigning consistent shift work schedules might be essential for reproductive health.

### 4.2. Strengths and Limitations

To our knowledge, this study is the first to quantify birth outcomes among female LEOs. We received maternal and child health data on over 400 pregnancies among 162 female LEOs from 32 states and provided detailed information on maternal characteristics and pregnancy outcomes. Additionally, this study directly responds to the call for more research addressing tactical athletes’ reproductive health concerns [22,54].

However, the study limitations include the self-reported nature of our study. Self-reported retrospective pregnancy outcomes, especially loss or terminations, may be sensitive information for some participants and may not accurately represent birth outcomes. However, the survey was anonymous and was advertised as such. While most LEOs reported an average of two to three pregnancies each, some females reported as many as nine. Researchers acknowledge that recollecting details about maternal health, fetal health, and job tasks during each pregnancy would be challenging and may not be entirely accurate. While we examined age, the strongest predictor of miscarriage, we did not control for other potentially confounding variables, such as previous miscarriages, or behavioral factors, such as smoking and alcohol use during pregnancy.

Further, we could not determine how representative this sample was of US female LEOs. Most of our sample identified as Caucasian; therefore, we may be missing critical reproductive health information from other ethnic groups. However, 79% of US LEOs (men and females) are Caucasian [7]. Complete racial and ethnic information on the entire US female LEO population is unavailable; therefore, exact statistics on the racial and ethnic representation of US female LEOs are not definitively known.

### 4.3. Future Recommendations

Our results justify the concern for the reproductive health of female LEOs. Future research should examine pregnancy complications, adverse birth outcomes, and potential mechanisms among female LEOs and other tactical occupations. Future epidemiological research should provide more insight into the magnitude or combinations of common LEO exposures and behavioral risk factors responsible for adverse reproductive health outcomes using regression or other statistical analytic approaches. Additional research is needed to examine the role of chronic stress and allostatic load in adverse birth outcomes for female LEOs. Future research should consider the reproductive health of dual law enforcement couples to determine if adverse birth outcomes are even greater among them. It would also be helpful to know the specific reproductive health concerns amongst female LEOs and if they impact recruitment and retention.

## 5. Conclusions

In conclusion, our study’s findings underscore the significant disparities in miscarriage and preterm birth rates among female law enforcement officers compared to large general-population studies and established benchmarks. These disparities persist even after adjusting for age, highlighting the unique challenges that female LEOs may face in their reproductive health journeys. The data reveal that age-adjusted miscarriage rates among female LEOs are notably higher, especially in those aged 35 and older, compared to the general population and international studies. Similarly, our study identified a substantial elevation in preterm birth rates, likely influenced by occupational exposures and high job stress experienced by these officers during their pregnancies.

These findings emphasize the importance of addressing occupational risks, providing the appropriate support, and considering consistent shift work schedules to safeguard the reproductive health of female law enforcement officers. Further research and policy considerations are warranted to mitigate these disparities and ensure the well-being of this vital segment of our workforce.

## 6. Policy Recommendations

To improve pregnancy outcomes for female law enforcement officers, we recommend the following:

1. Mandating reproductive health education for LEOs and supervisors, covering modifiable behavioral factors like caffeine intake, drug use, alcohol use, smoking, and BMI.
2. Implementing policies such as offering shifts lasting less than 10 h, removing individuals from night shift duty, and maintaining a consistent work schedule of 40 h or less per week during the first 12 weeks of fetal gestation.
3. Providing ongoing stress management tools to female LEOs to enhance pregnancy outcomes.
4. Counseling females of childbearing age about potential miscarriage risks associated with occupational exposures like shift work, high occupational stress, and environmental factors.
5. Monitoring all pregnant LEOs closely for gestational hypertension, promoting a healthy pre-pregnancy body weight, and educating them about the risks of preterm birth.
6. Recognizing that stress management for female LEOs may contribute to minimizing reproductive harm and increasing female representation in law enforcement.

## Figures and Tables

**Table 1:** Participant demographic characteristics (N = 162).

Domain	n (%)
Age	
18–30	15 (9.3)
31–40	68 (42.0)
41–49	49 (30.2)
50 and older	20 (12.3)
Missing	10 (6.2)
Number of pregnancies	
One pregnancy	40 (24.5)
Two pregnancies	58 (35.6)
Three pregnancies	36 (22.1)
Four pregnancies	16 (9.8)
Five pregnancies	6 (3.7)
Six pregnancies	5 (3.1)
Seven pregnancies	1 (0.6)
Nine pregnancies	1 (0.6)
Income	
USD 75,000 or less	23 (14.2)
USD 75,000 to USD 100,000	32 (19.8)
Greater than USD 100,000	88 (54.3)
Prefer not to answer/missing	19 (11.7)
Education	
Some high school	1 (0.6)
High school graduate or GED	2 (1.2)
Some college or technical school	51 (31.5)
College graduate	69 (42.9)
Advanced degree	26 (16.0)
Missing	13 (8.0)

**Table 2:** Job-specific demographic characteristics (N = 162).

Characteristic	n (%)
Years of law enforcement service	
5 years or less	18 (11.1)
6–10 years	30 (18.5)
11–15 years	45 (27.8)
16–19 years	25 (15.4)
20+ years	44 (27.2)
Rank	
Captain	2 (1.2)
Chief/Sheriff	3 (1.9)
Corporal	4 (2.5)
Lieutenant	16 (9.9)
Sergeant	29 (17.9)
Detective/Investigator	31 (19.1)
Officer (police, patrol, constable, or deputy sheriff)	77 (47.5)
Department classification	
Urban	81 (50.0)
Suburban	50 (30.9)
Rural	27 (16.7)
Don’t know	4 (2.4)

**Table 3:** Frequency of specific outcomes by number of pregnancy for female LEOs < 35 years and ≥ 35 years of age.

Pregnancy	Age Category	Livebirth	Miscarriage	Termination
First	Age < 35, n = 150	111 (74.0%)	26 (17.3%)	13 (8.7%)
	Age ≥ 35, n = 12	5 (41.7%)	7 (58.3%)	0
Second	Age < 35, n = 98	83 (84.7%)	12 (12.2%)	3 (3.1%)
	Age ≥ 35, n = 20	15 (75.0%)	4 (21.1%)	1 (5.3%)
Third	Age < 35, n = 41	30 (73.2%)	8 (19.5%)	3 (7.3%)
	Age ≥ 35, n = 21	18 (85.7%)	3 (15.0%)	0
Fourth–Ninth	Age < 35, n = 32	17 (53.1%)	5 (15.6%)	10 (31.3%)
	Age ≥ 35, n = 20	14 (70.0%)	4 (20.0)	2 (10.0%)

**Table 4:** Pregnancy and live birth outcomes by number of pregnancies.

Conditions	Pregnancy			
	First n (%)	Second n (%)	Third n (%)	Fourth–Ninth n (%)
Known maternal condition	(n = 22)	(n = 20)	(n = 15)	(n = 16)
High blood pressure	14 (63.6)	13 (65.0)	8 (53.3)	10 (62.5)
Diabetes	8 (36.4)	7 (35.0)	7 (46.7)	6 (37.5)
Known baby condition	(n = 70)	(n = 48)	(n = 31)	(n = 31)
Baby weighed less than 2.5 kg	11 (15.7)	4 (8.3)	3 (9.7)	1 (3.2)
Baby born > 3 weeks before due date	21 (30.0)	12 (25.0)	12.0 (38.7)	2 (6.5)
Baby had jaundice at birth	36 (51.4)	29 (60.4)	13 (41.9)	9 (29.0)
Baby had failed to thrive	2 (2.9)	3 (6.3)	3 (9.7)	19 (61.3)

## Data Availability

Data are available upon request to ainslie@drainsliek.com.
